# Supplementary material for: Synthesis and Electrochemical Performance of the Orthorhombic V2O5·nH2O Nanorods as Cathodes for Aqueous Zinc Batteries
Source: Nanomaterials (Basel). 2022 Jul 23;12(15):2530. doi: 10.3390/nano12152530 (PMC9332479; doi:10.3390/nano12152530)
Supplement: Supplementary file 1 [file nanomaterials-12-02530-s001.zip › nanomaterials-1819487-supplementary.pdf]

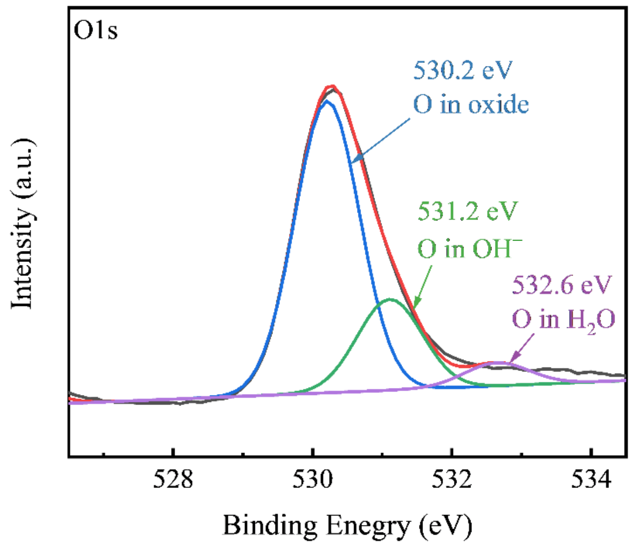

Figure S1. O 1s XPS spectrum in $V_2O_5{\cdot}nH_2O$ nanorods.

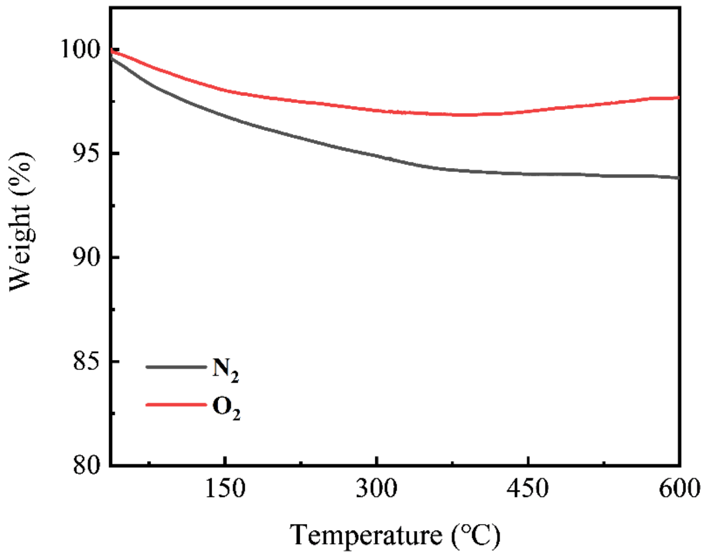

Figure S2. TG curves of the $V_2O_5{\cdot}nH_2O$ nanorods under O_2 and N_2 atmospheres.

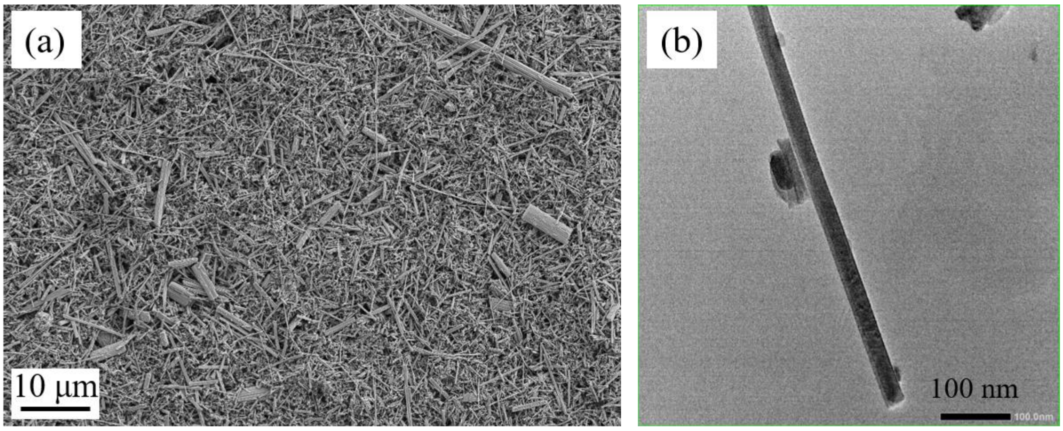

(c)

Element	Weight (%)	Atomic (%)
**O K**	43.54	71.06
**V K**	56.46	28.94
**Total**	100.00	100.00

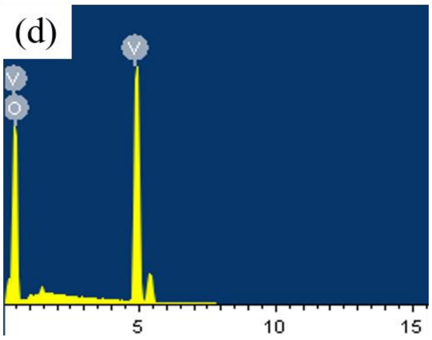

Figure S3. (**a**) SEM, (**b**) TEM images of $V_2O_5{\cdot}nH_2O$ nanorods at low magnification and (**c**,**d**) elemental results from EDX measurement.

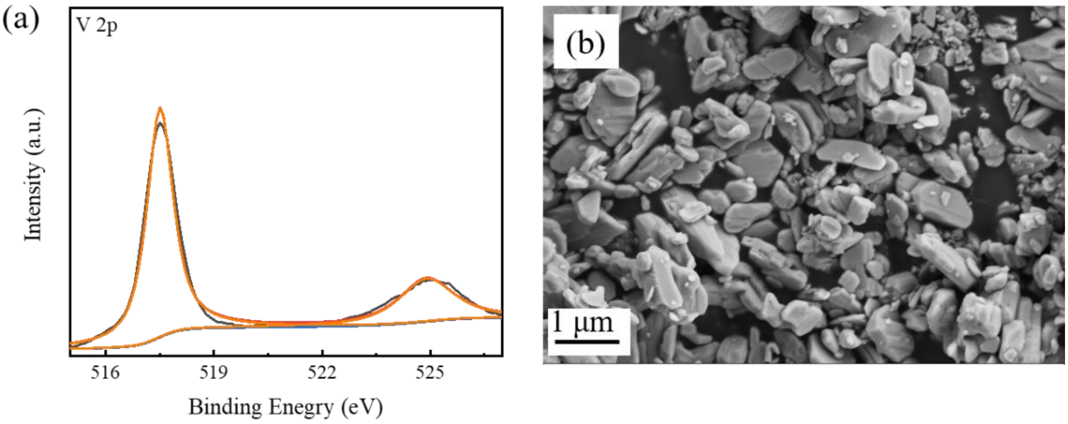

Figure S4. (**a**) V 2p XPS spectrum and (**b**) SEM image of commercial V_2O_5 powders.

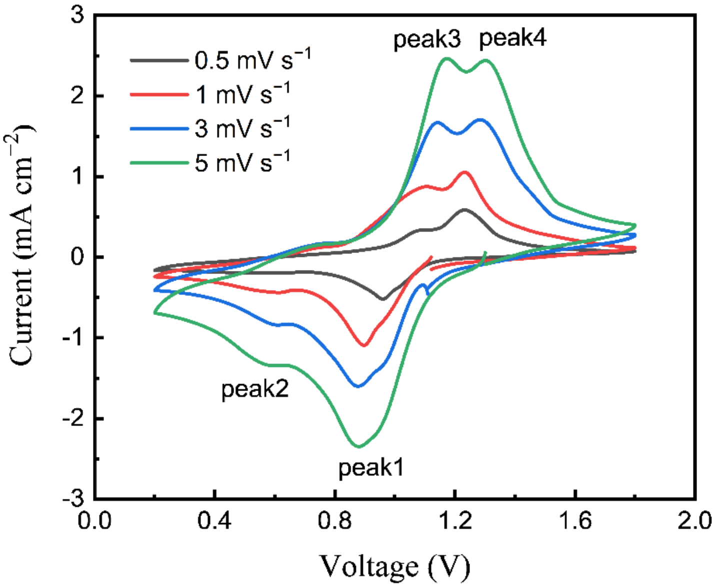

Figure S5. CV curves of $V_2O_5{\cdot}nH_2O$ nanorods at different scan rates.

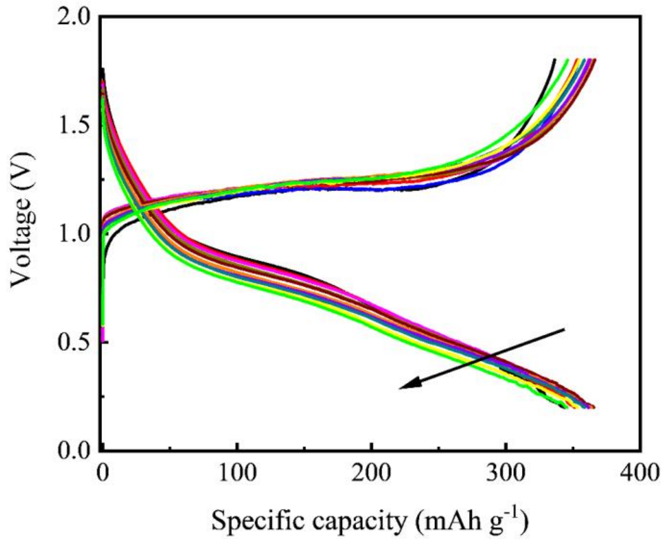

Figure S6. Selected discharge-charge patterns for $V_2O_5{\cdot}nH_2O$ nanorods cathode at 1.0 A g^{-1} at 10th, 20th, 30th, 40th, 50th, 60th, 70th, 80th, 90th, 100th cycles.

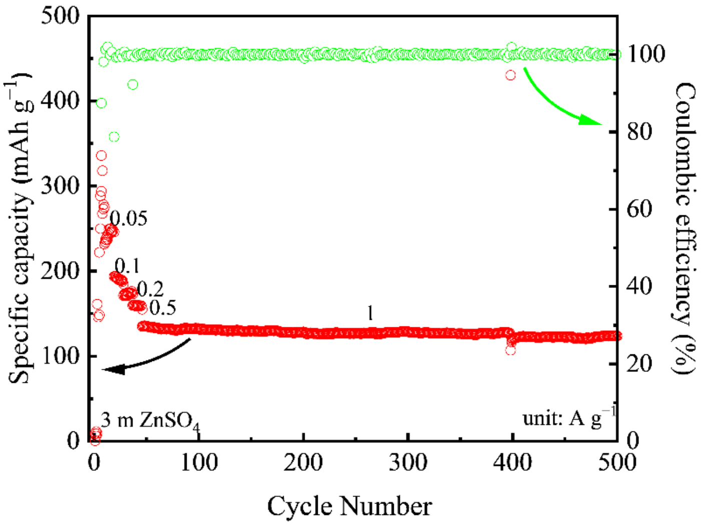

Figure S7. Cycling performance and corresponding Coulombic efficiencies of the commercial V_2O_5 powders at 1.0 A g^{-1} over 500 cycles.

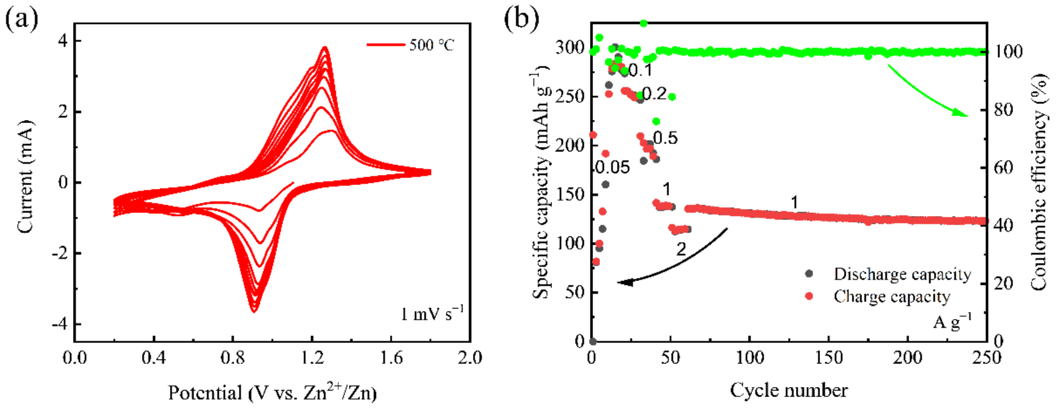

Figure S8. CV curves (**a**) and cycling performance with Coulombic efficiencies (**b**) of V_2O_5 nanorods annealed at 500 °C.

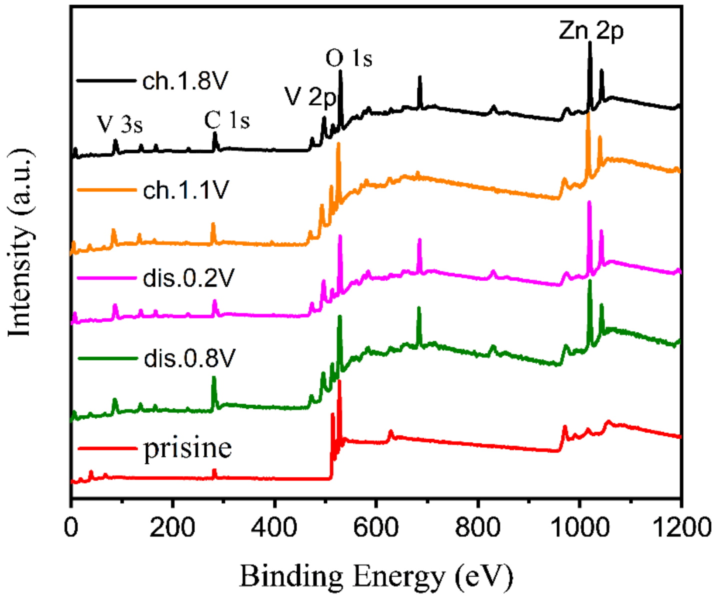

Figure S9. The full XPS spectra of the $V_2O_5{\cdot}nH_2O$ nanorods at different charge–discharge states.

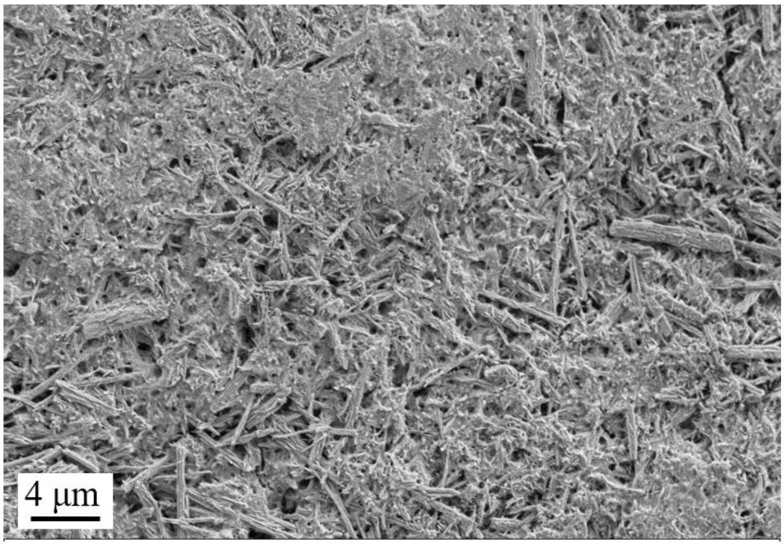

Figure S10. SEM image of $V_2O_5{\cdot}nH_2O$ nanorods after 200 cycles at low magnification.

Table S1. Electrochemical performance of the reported orthorhombic V_2O_5 materials in aqueous Zn batteries.

Materials	Capacity	Cycling	Electrolyte/Zn	Ref.
**V_2O_5 nanoparticles**	224 mAh g^{-1} at 0.1 A g^{-1}	120 mAh g^{-1} at 1 A g^{-1} after 400 cycles	3M $ZnSO_4$/ Zn foil	[24]
**Ball-milled commercial V_2O_5**	453 mAh g^{-1} at 0.1 A g^{-1}	372 mAh g^{-1} at 5 A g^{-1} after 4000 cycles	3 M $Zn(CF_3SO_3)_2$/ Zn foil	[23]
**V_2O_5 hollow spheres**	360 mAh g^{-1} at 0.1 A g^{-1}	150 mAh g^{-1} at 0.5 A g^{-1} after 265 cycles	saturated $ZnSO_4$/ Zn foil	[35]
**V_2O_5@CNTs**	300 mAh g^{-1} at 0.1 A g^{-1}	152 mAh g^{-1} at 5 A g^{-1} after 6000 cycles	saturated $ZnSO_4$/ Zn foil	[S1]
**Porous V_2O_5 nanofibers**	265 mAh g^{-1} at 0.02 A g^{-1}	166 mAh g^{-1} at 0.59 A g^{-1} after 500 cycles	3 M $Zn(CF_3SO_3)_2$/ Zn foil	[S2]
**V_2O_5 nano paper**	375 mAh g^{-1} at 0.5 A g^{-1}	335 mAh g^{-1} at 1.0 A g^{-1} after 100 cycles	2 M $ZnSO_4$/ Zn metal pellet	[36]
**V_2O_5 nanospheres**	262 mAh g^{-1} at 1.0 A g^{-1}	110 mAh g^{-1} at 1.0 A g^{-1} after 80 cycles	2 M $ZnSO_4$/ Zn metal plate	[37]
**$V_2O_5 \cdot nH_2O$ nanorods**	507 mAh g^{-1} at 0.05 A g^{-1}	230 mAh g^{-1} at 1.0 A g^{-1} after 500 cycles	3 m $ZnSO_4$/ Zn foil	This work
**$V_2O_5 \cdot nH_2O$ nanorods**	330 mAh g^{-1} at 1.0 A g^{-1}	156 mAh g^{-1} at 1.0 A g^{-1} after 100 cycles	3 m $ZnSO_4$/ Zn powder	This work

Supplementary References

1. Chen, H.; Qin, H.; Chen, L.; Wu, J.; Yang, Z. V_2O_5@CNTs as cathode of aqueous zinc ion battery with high rate and high stability. *J. Alloys Compd.* **2020**, *842*, 155912. https://doi.org/10.1016/j.jallcom.2020.155912.
2. Chen, X.; Wang, L.; Li, H.; Cheng, F.; Chen, J. Porous V_2O_5 nanofibers as cathode materials for rechargeable aqueous zinc-ion batteries. *J. Energy Chem.* **2019**, *38*, 20–25. https://doi.org/10.1016/j.jechem.2018.12.023.
